# Novel large deletion involving *EVC* and *EVC2* in Ellis–van Creveld syndrome

**DOI:** 10.1038/s41439-022-00190-0

**Published:** 2022-05-17

**Authors:** Hiroki Sato, Kenichi Suga, Masashi Suzue, Yukako Honma, Yasunobu Hayabuchi, Shunsuke Miyai, Hiroki Kurahashi, Ryuji Nakagawa

**Affiliations:** 1 Department of Pediatrics, Tokushima University Hospital, Kuramotocho, Tokushima, Japan; 2 Division of Molecular Genetics, Institute for Comprehensive Medical Science, Fujita Health University, Toyoake, Japan

**Keywords:** Congenital heart defects, Rare variants

## Abstract

Ellis–van Creveld syndrome is an autosomal recessive skeletal dysplasia that is characterized by thoracic hypoplasia, polydactyly, oral abnormalities, and congenital heart disease. It is caused by pathogenic variants in the *EVC* or *EVC2* genes. We report a case of a newborn with a compound heterozygous variant comprising NM_147127.5: c.1991dup:[p.Lys665Glufs*10] in the *EVC2* gene and a novel large deletion involving exon 1 in *EVC* and exons 1–7 in *EVC2*.

Ellis–van Creveld syndrome (EVC) (OMIM #225500) is an autosomal recessive disease that is characterized by thoracic hypoplasia, short ribs, short limbs, postaxial polydactyly, dysplastic teeth and nails, and congenital heart disease^1^. EVC is caused by variants of the EVC ciliary complex subunit 1 gene (*EVC*) (OMIM #604831) and EVC ciliary complex subunit 2 gene (*EVC2*) (OMIM #607261), which are located on chromosome 4p16. The encoded proteins constitute a complex that is expressed in the basal primary cilia. Biallelic loss-of-function variants in *EVC* or *EVC2* lead to primary ciliary dysfunction, namely, skeletal ciliopathies^2^.

With an overall prevalence of 0.7 per 100,000 live births^3^, EVC is rare. However, it is more frequent among the Amish community in Lancaster County, Pennsylvania, USA, and some Arab populations, where it occurs at a prevalence of 5 per 1000 and 5.2 per 100,000 live births, respectively^4^. For the Amish population, there are some genetically notable characteristics. First, the Amish population is a closed group that migrated from Germany in the 18th century. Second, they adhere to strict marriages. Past research has shown that both parents of 50 cases of EVC can be traced back to one couple, Mr. and Mrs. Samuel King^5^. This proves the founder effect and autosomal recessive pattern.

Here, we describe a newborn with an EVC-associated truncating compound heterozygous mutation that comprises a novel large deletion in *EVC* and *EVC2* and a previously reported frameshift variant in *EVC2*.

A male was born to nonconsanguineous healthy parents—a 28-year-old father and a 28-year-old primipara (1 gravida and 0 para)—at 40 weeks of gestational age after oxytocin-induced labor due to uterine inertia. No fetal abnormalities were noted on fetal ultrasonography. Fetal deceleration and meconium staining of the amniotic fluid occurred during delivery. After birth, neonatal resuscitation with mask bagging was required for neonatal asphyxia. Apgar scores were 4, 6, and 8 at 1, 5, and 10 min, respectively.

On physical examination at birth, the newborn had bilateral polydactyly (Fig. 1A), natal teeth, and mesomelic shortening of the limbs. The birth weight was 3230 g (+0.4 SD), birth length was 50.0 cm (+0.3 SD), and head circumference was 35.0 cm (+1.2 SD) (SDs based on Japanese neonatal standards)^6^. Chest X-ray revealed bell-shaped thoracic hypoplasia, left clavicle fracture, and pulmonary air leakage in the right lung (Fig. 1B). Cardiac ultrasonography showed a complete atrioventricular septal defect (Rastelli type A) and bidirectional shunting (right-to-left dominant) at the ductus arteriosus and massive tricuspid valve regurgitation, suggesting persistent pulmonary hypertension of newborn syndrome. Because of his lung hypoplasia and multiple congenital anomalies, the patient was transferred from the secondary neonatal intensive care unit to the tertiary neonatal intensive care unit at Tokushima University Hospital.

**Fig. 1:**
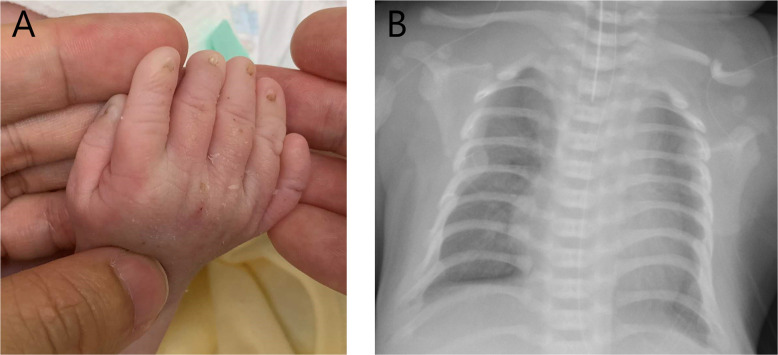
Images of the patient. **A** Image of the patient’s right hand. He had bilateral polydactyly and nail dysplasia. **B** Thoracic image of the patient. He shows thoracic hypoplasia and a bell-shaped thorax. The pneumothorax in the right lung was due to artificial ventilation. His left clavicle was fractured during delivery.

On admission, mechanical ventilation with high-frequency oscillation and inhaled nitric oxide for persistent pulmonary hypertension of the newborn were initiated. At 3 days of age, inhaled nitric oxide and sedative agents were discontinued. At 4 days of age, he was extubated and switched to a high-flow nasal cannula. At 25 days of age, he began receiving nasal oxygen. At 43 days, he was discharged with home oxygen therapy (1 L/min). Due to inadequate oral intake, he required tube feeding since he was 2 months old. At 12 months, he showed severe growth delay (body weight, –3.2 SD; height, –2.9 SD) and mild developmental delay (creeping along the floor) and still required home oxygen therapy and tube feeding. His chromosomal karyotype was 46, XY with inv(9)(p11q13) (normal benign variant).

We provided genetic counseling for his parents and obtained written informed consent for genetic testing. We obtained blood samples from the patient and his parents. Targeted panel sequencing for genomic DNA extracted from peripheral blood lymphocytes was performed using NextSeq2000 (Illumina, San Diego, CA, USA) at Kazusa DNA Research Institute. Reads were aligned to the human genome sequence assembly GRCh38/hg38, and the obtained mean read depths were approximately 250. To identify pathogenic single nucleotide variants (SNVs), we excluded sequence variants with low allele frequencies (>0.01) in dB SNP (https://www.ncbi.nlm.nih.gov/snp/), Genome Aggregation Database (gnomAD, https://gnomad.broadinstitute.org/), and Japanese Multi Omics Reference Panel (jMorp, https://jmorp.megabank.tohoku.ac.jp/202008/). Sequencing of the *EVC* and *EVC2* genes revealed two heterozygous variants, NM_147127.5:c.1991dup (exon 13):[p.Lys665Glufs*10] at *EVC2* and NM_153717.3:c.2645G > T (exon 18):[p.Gly882Val] at *EVC*. In addition, quantitative analysis suggested a large deletion encompassing exon 1 of *EVC* and exons 1–7 of *EVC2*. The consequent Sanger sequencing confirmed the c.1991dup mutation in *EVC2* and the c.2645G > T mutation in *EVC* (Fig. 2A), which were derived from the patient’s father. Multiplex ligation-dependent probe amplification confirmed the large deletion comprising exon 1 in *EVC* and exons 1–7 in *EVC2*, which was the same as in his mother (Fig. 2B). We evaluated the pathogenicity of these variants in accordance with the guidelines of the American College of Medical Genetics and Genomics^7^. NM_147127.5:c.1991dup is a null variant (frameshift) in the *EVC2* gene for which loss of function is a known mechanism associated with EVC (PVS1)^8^. This variant was not found in population databases, including gnomAD (https://gnomad.broadinstitute.org), dbSNP (https://www.ncbi.nlm.nih.gov/snp/), jMorp (https://jmorp.megabank.tohoku.ac.jp/202109/), and TogoVar (https://togovar.biosciencedbc.jp/) (PM2). A pathogenic variant was detected in trans with a pathogenic variant (PM3). The pathogenic computational verdict from mutation taster (http://www.mutationtaster.org/) based on 1 pathogenic prediction vs. no benign predictions was PP3. As a result, c.1991dup was determined to be pathogenic (1PVS, 2PM, 1PP) according to the guidelines of the American College of Medical Genetics and Genomics^7^. NM_153717.3:c.2645G > T was not found in the above listed population databases (PM2). A pathogenic variant (deletion) was detected in trans with a pathogenic variant (PM3). c.2645G > T was also not found in ClinVar (https://www.ncbi.nlm.nih.gov/clinvar/). The position is not conserved (phyloP100way = –0.111, which is less than 3.46). c.2645G > T was determined to be tolerated or neutral according to 24 in silico predictive databases, including M-CAP (http://bejerano.stanford.edu/mcap/), PROVEAN CADD (score, 9.197; https://cadd.gs.washington.edu/snv), and PolyPhen-2 (http://genetics.bwh.harvard.edu/pph2/)(BP4). As a result, c.2645G > T was determined to be a variant of uncertain significance (two moderate [PM2, PM3] and one BP4).

**Fig. 2:**
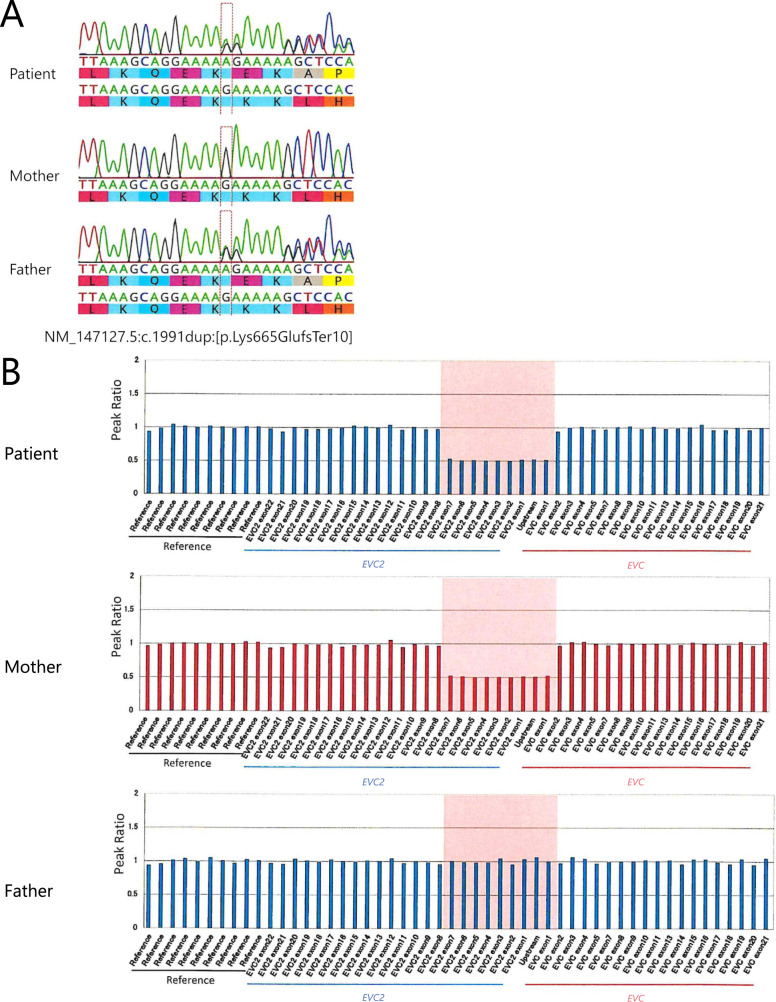
The results of genetic analysis. **A** The results of Sanger sequencing. The patient and his father share a heterozygous frameshift variant of EVC2: c.1991dup:[p.Lys665GlufsTer19]. **B** Results of multiplex ligation-dependent probe amplification. The patient and his mother both have the deletion encompassing exon 1 in *EVC* and exons 1–7 in *EVC2* (region marked by pink shading).

In our patient, we identified the compound heterozygous variant c.1991dup (exon 13) in *EVC2* and a large deletion involving exon 1 in *EVC* and exons 1–7 in *EVC2*. The Human Gene Mutation Database (http://www.hgmd.cf.ac.uk/ac/index.php) includes 8 gross deletions in *EVC* and 6 gross deletions in *EVC2*. Three pedigrees of EVC with large deletions in *EVC* and *EVC2* as well as in the contiguous genes *C4orf6* and *STK32B* have been reported^9,10^. However, these patients showed borderline intelligence, which is not typical for EVC and probably depends on *C4orf6* or *STK32B*. To our knowledge, the present deletion is a novel contiguous deletion that is limited to *EVC* and *EVC2*. *EVC* and *EVC2* form a head-to-head configuration on 4p16.2. They are separated by 2.6 kb of genomic sequence and share a common transcriptional promoter^11^. Therefore, in the present case, the large deletion encompassing exon 1 of *EVC* and exons 1–7 of *EVC2* included the shared promoter of *EVC* and *EVC2*. Both alleles of our patient’s *EVC2* gene and one allele of his *EVC* gene are nonfunctional due to the promoter deletion and frameshift variant of the gene.

Ohashi et al.^8^ reported a lethal thoracic hypoplasia case of EVC that had the compound heterozygous variants c.1991dup and c.871-3C > G (intron 7) on *EVC2*. However, in the present case, thoracic hypoplasia was mild, and the patient was able to be discharged with home oxygen therapy. Thus, the phenotypes of EVC do not always match the genotypes of *EVC* and/or *EVC2*.

EVC is a type of ciliopathy. In wild-type mice, Evc, Evc2, and Smo form a complex, which is located in the EvC zone of primary cilia in the presence of Sonic Hedgehog (Shh) ligands^12^. This complex promotes Hedgehog (Hh) signaling, thereby promoting transcription. In the case of EVC, the Hh signal is not transmitted even in the presence of Shh ligands^13^. Shh^–/–^ mice are nonviable, and Shh^–/–^ mouse embryos display several heart defects, including atrioventricular septal defects^14,15^. Hh-responsive cells in the growth plate comprise osteogenic progenitors that can directly differentiate into osteoblasts^16^. Shh signaling regulates the formation of various tooth components, including enamel, dentin, cementum, and various soft tissues^17^.

Other skeletal ciliopathies with overlapping features include short rib polydactyly syndrome^18^, Jeune syndrome^19^, and cranioectodermal dysplasia^2,20^, all of which show thoracic hypoplasia and short ribs. McKusick–Kaufman syndrome is characterized by polydactyly and congenital heart disease^18^, and Weyers acrofacial dysostosis is characterized by dental abnormalities, nail dystrophy, and polydactyly^21^. Identifying gene alterations is important because these diseases are difficult to differentiate based on their clinical phenotypes^2^.

Concerning our patient’s family, the recurrence risk for subsequent pregnancies is 25%, and we have provided this genetic information to the patient’s parents. Although the patient had not been diagnosed in the fetal period, the fetus of any consequent pregnancy can be diagnosed prenatally according to the existence of polydactyly, thoracic hypoplasia, and cardiac malformations, such as atrioventricular septal defects.

This report has several limitations. First, we did not perform any confirmatory array comparative genomic hybridization to identify the deletion point. Second, the follow-up period was short. Our patient had mild developmental delay, which should be continued to be monitored.

In conclusion, we describe a novel large deletion involving both *EVC* and *EVC2*. The phenotype of this variant seems to be typical for EVC.

## Data Availability

The relevant data from this Data Report are hosted at the Human Genome Variation Database at 10.6084/m9.figshare.hgv.3157.
